# Insights Into the Function and Clinical Application of HDAC5 in Cancer Management

**DOI:** 10.3389/fonc.2021.661620

**Published:** 2021-06-10

**Authors:** Jun Yang, Chaoju Gong, Qinjian Ke, Zejun Fang, Xiaowen Chen, Ming Ye, Xi Xu

**Affiliations:** ^1^ Department of Orthopedic Surgery, Sanmen People’s Hospital of Zhejiang Province, Sanmenwan Branch of the First Affiliated Hospital, College of Medicine, Zhejiang University, Sanmen, China; ^2^ Central Laboratory, The Municipal Affiliated Hospital of Xuzhou Medical University, Xuzhou, China; ^3^ Central Laboratory, Sanmen People’s Hospital of Zhejiang Province, Sanmenwan Branch of the First Affiliated Hospital, College of Medicine, Zhejiang University, Sanmen, China; ^4^ Department of Pathophysiology, Zunyi Medical University, Zunyi, China; ^5^ Department of General Surgery, Sanmen People’s Hospital of Zhejiang Province, Sanmenwan Branch of the First Affiliated Hospital, College of Medicine, Zhejiang University, Sanmen, China; ^6^ Department of Pathology, The Second Affiliated Hospital, Zhejiang University School of Medicine, Hangzhou, China

**Keywords:** HDAC5, cancer, biological function, clinical application, biomarker

## Abstract

Histone deacetylase 5 (HDAC5) is a class II HDAC. Aberrant expression of HDAC5 has been observed in multiple cancer types, and its functions in cell proliferation and invasion, the immune response, and maintenance of stemness have been widely studied. HDAC5 is considered as a reliable therapeutic target for anticancer drugs. In light of recent findings regarding the role of epigenetic reprogramming in tumorigenesis, in this review, we provide an overview of the expression, biological functions, regulatory mechanisms, and clinical significance of HDAC5 in cancer.

## Introduction

Covalent modification of chromatin regulates gene transcription during cell differentiation; this includes histone acetylation and deacetylation, which modulate the binding of transcription factors to DNA. Modifications can occur at various sites, including the N-terminal amino acid residues of histones H3 and H4, and the N- and C-terminal amino acid residues of histones H2A, H2B, and H1. Histone acetylation of the ϵ-amino group of lysine, one of the earliest and most common posttranslational modifications of histones, is regulated by histone acetyltransferases and histone deacetylases (HDACs) (1–5).

The HDAC family has 18 members that can be divided into 4 classess based on their structure and function (6, 7). Class I includes HDAC1-3 and 8; class II includes HDAC4-7, 9, and 10; and class III includes sirtuin enzymes (SIRT 1-7). These 3 classes constitute the classical HDACs. Class IV comprises only HDAC11, which is structurally distinct from the other HDACs. Class II HDACs are further divided into class IIa (HDAC4, 5, 7, and 9) and class IIb (HDAC6 and 10) based on their subcellular localization and expression pattern (8).

Class IIa HDACs have a high degree of homology to yeast hda1 (HDAC-1). In addition to the C-terminal catalytic core, all class IIa HDACs have a conserved N-terminal extension that can bind to transcription factors and the chaperone protein 14-3-3. For example, when phosphorylated by calcium/calmodulin dependent protein kinase (CaMK) or protein kinase D (PKD), class IIa HDACs bind to 14-3-3, inducing its disaggregation from the transcription factor myocyte specific enhancer factor 2 (MEF2) and bringing about its shuttling from the nucleus to the cytoplasm (9).

As a class IIa HDAC, HDAC5 not only catalyzes the deacetylation of nuclear histones, but also deacetylates or forms complexes with other proteins in various physiologic contexts including nerve regeneration and neuronal apoptosis (10, 11), glucose metabolism (12), and insulin resistance (13). Cancer is one of the leading causes of death worldwide, and the identification of further specific molecular markers of cancer will be useful for early diagnosis, therapeutic targeting, and treatment response monitoring. This review highlights the current state of knowledge of the roles and clinical significance of HDAC5 in tumorigenesis.

## Structure and Distribution of HDAC5

### Structure of HDAC5

HDAC5 was first identified in the mouse genome in 1999 (14). The gene encoding HDAC5 (also known as HD5 or NY-CO-9) in humans is located on chromosome 17q21 and spans 39,138 bp, comprising 26 exons. The protein consists of 1122 amino acids, has a molecular weight of 121.9 kDa, and has C-terminal deacetylase and N-terminal adapter domains. The former domain, which is also referred to as the HDAC domain, contains a nuclear export sequence(NES) and is highly conserved (80% homology) across class IIa HDACs; it is composed of 400-450 amino acids and shares 53% sequence similarity with yeast hda1 (15). The conserved 450-600 amino acid N-terminal adaptor domain has a nuclear localization sequence (NLS) and binds to transcription factors such as C-terminal-binding protein (CtBP), MEF2, and heterochromatin P1 (HP1) (3).

HDAC5 also has a flexible zinc-binding element outside the catalytic core. This element contains highly conserved cysteine and histidine residues that form a hydrophobic pocket near the substrate-binding channel. It may mediate substrate recognition and regulate enzymatic activity and interactions with other proteins (16). Moreover, HDAC5 also has a conserved histidine near the first zinc-binding site whose side chain turns outward and is far away from the catalytic active site (17). Thus, while HDAC5 by itself has no deacetylase activity, it can be induced by interaction with HDAC3 through the silencing mediator of retinoic acid and thyroid hormone receptor (SMRT)/nuclear receptor corepressor (NCoR) coinhibition complex (18). In other words, HDAC5 regulates gene expression by binding to transcription factors through its N terminal adapter domain and targeting the SMRT/NCoR-HDAC3 complex to a specific subcellular location through its C-terminal deacetylase domain.

### Distribution of HDAC5

HDAC5 protein is expressed in lung, brain, myocardium, skeletal muscle, and placenta, and accumulating evidence indicates that it has variable expression and functions in different types of tumor: HDAC5 is overexpressed in breast cancer (19, 20), hepatocellular carcinoma (HCC) (21), lung cancer (22), pancreatic neuroendocrine cancer(pNET) (23) and colorectal cancer(CRC) (24). In contrast, although HDAC5 was shown to induce tissue invasion of gastric cancer cells (25), gene expression profiles of histone modifiers indicate that HDAC5 is downregulated in gastric cancer (26) (**Table 1**). These conflicting findings imply that HDAC5 exhibits dual functions in cancer development. Furthermore, HDAC5 mRNA and protein have been detected in the blood of patients with CRC (32, 33) and breast cancer (34), but not in that of healthy subjects or patients with nonrecurrent cancer, suggesting that circulating HDAC5 may serve as a potential biomarker for cancer diagnosis and prognosis.

**Table 1:** The expression of HDAC5 in various tumors.

Tumor types	Expression status	Detection methods	References
Breast cancer	Up-regulation	IHC	(20)
		IHC, qRT-PCR	(19)
Hepatocellular carcinoma	Up-regulation	IHC	(21)
Lung cancer	Up-regulation	Western Blot, qRT-PCR	(22)
		qRT-PCR	(27)
Melanoma	Up-regulation	IHC, Western Blot	(28)
Pancreatic neuroendocrine cancer	Up-regulation	IHC	(23)
Colorectal cancer	Up-regulation	qRT-PCR	(24)
Glioma	Up-regulation	Western Blot, qRT-PCR	(29)
Osteosarcoma	Up-regulation	Western Blot, qRT-PCR	(30)
Wilms’ tumor	Up-regulation	Western Blot, qRT-PCR	(31)
Gastic cancer	Down-regulation	qRT-PCR	(26)

## Regulation of HDAC5

### Posttranslational Modification of HDAC5

**Figure 1:**
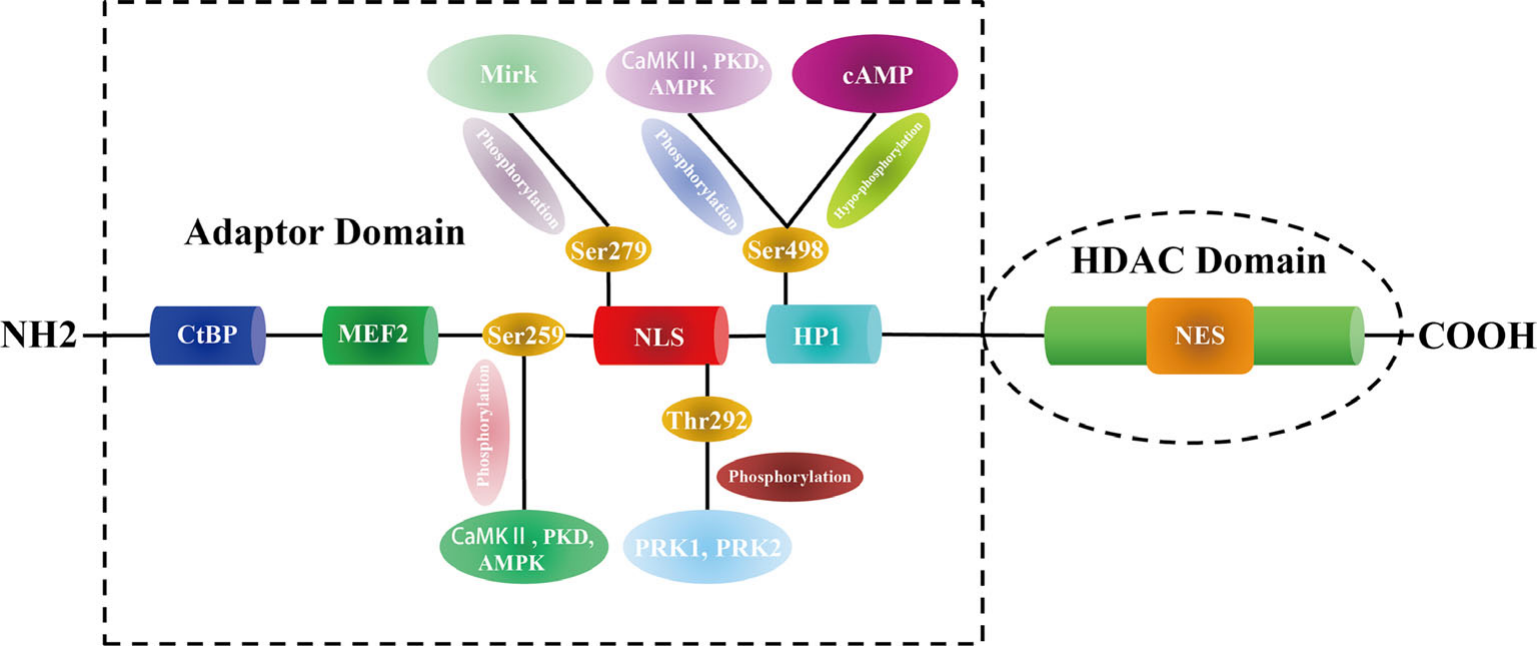
The structure and modification sites of HDAC5.

Proteomics analysis combined with phosphomutant screening has revealed that there are at least 17 phosphorylation sites in the HDAC5 functional domains (35), highlighting that HDAC5 can be phosphorylated at multiple conserved residues by a variety of protein kinases. For example, PKD, CaMK II, and AMPK phosphorylate Ser259 and Ser498 on both sides of the HDAC5 NLS, which promotes the binding of 14-3-3 to HDAC5 and its shuttling from the nucleus to the cytoplasm (36–38). In myocyte-like cells, cAMP signaling prevents 14-3-3 binding to HDAC5 by bringing about Ser498 hypophosphorylation (39). Protein kinase C-related kinase 1/2 (PRK1/2) phosphorylate HDAC5 at Thr292 in the NLS, promoting its binding to 14-3-3 and preventing HDAC5 nuclear entry (40). Additionally, minibrain-related kinase (Mirk) phosphorylates HDAC5 at Ser279, preventing its translocation from the cytoplasm to the nucleus (41) (**Figure 1**).

### Non-Coding RNAs Modification to HDAC5

HDAC5 expression is regulated by miRNAs. miR-2861 was the first reported miRNA to directly regulate HDAC5, which it does by binding to the HDAC5 mRNA coding sequence (42). miR-9 suppresses HDAC5 activity (43) and inhibits the translation of HDAC5 transcript by binding to the 3′ untranslated region (44). Other miRNAs known to regulate HDAC5 are miR-124 (44, 45), miR-125a-5p (46), miR-589-5p (27), and miR-217 (47). Many miRNAs targeting HDAC5 have been identified using target prediction software (**Supplementary Table 1**), although most of these require experimental validation.

**Figure 2:**
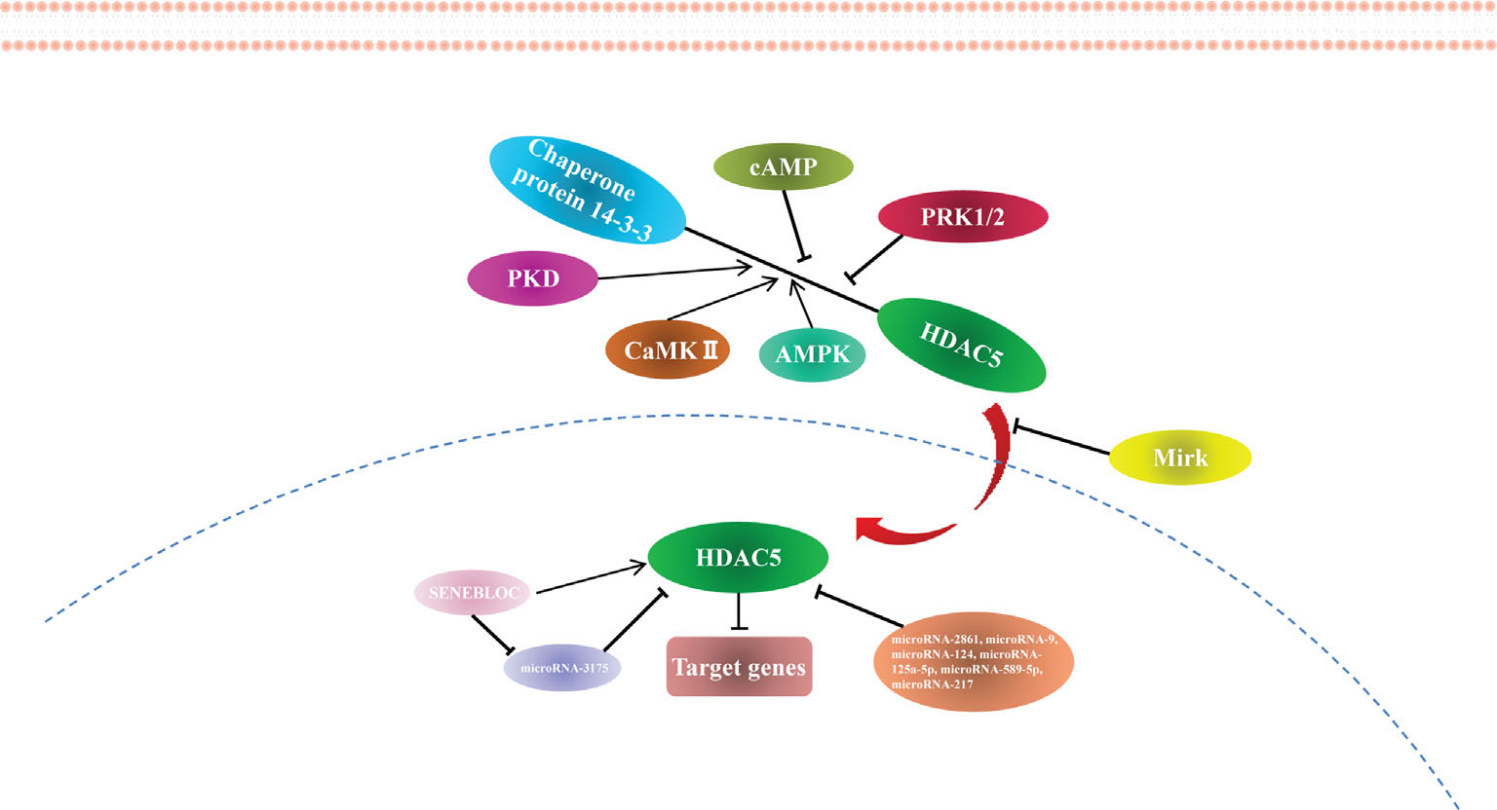
The regulation of HDAC5.

According to the competing endogenous RNA (ceRNA) hypothesis, long noncoding RNAs (lncRNAs) inhibit miRNA function by acting as endogenous miRNA sponges (48). The lncRNA SENEBLOC acts as a sponge regarding miR-3175, thereby upregulating HDAC5 (49). Additional studies investigating the role of lncRNAs in HDAC5 regulation are currently underway. The findings to date indicate that HDAC5 is epigenetically modified and regulated at the transcriptional level (**Figure 2**), providing new avenues for HDAC5-based cancer therapy.

## HDAC5 in Cancer

**Figure 3:**
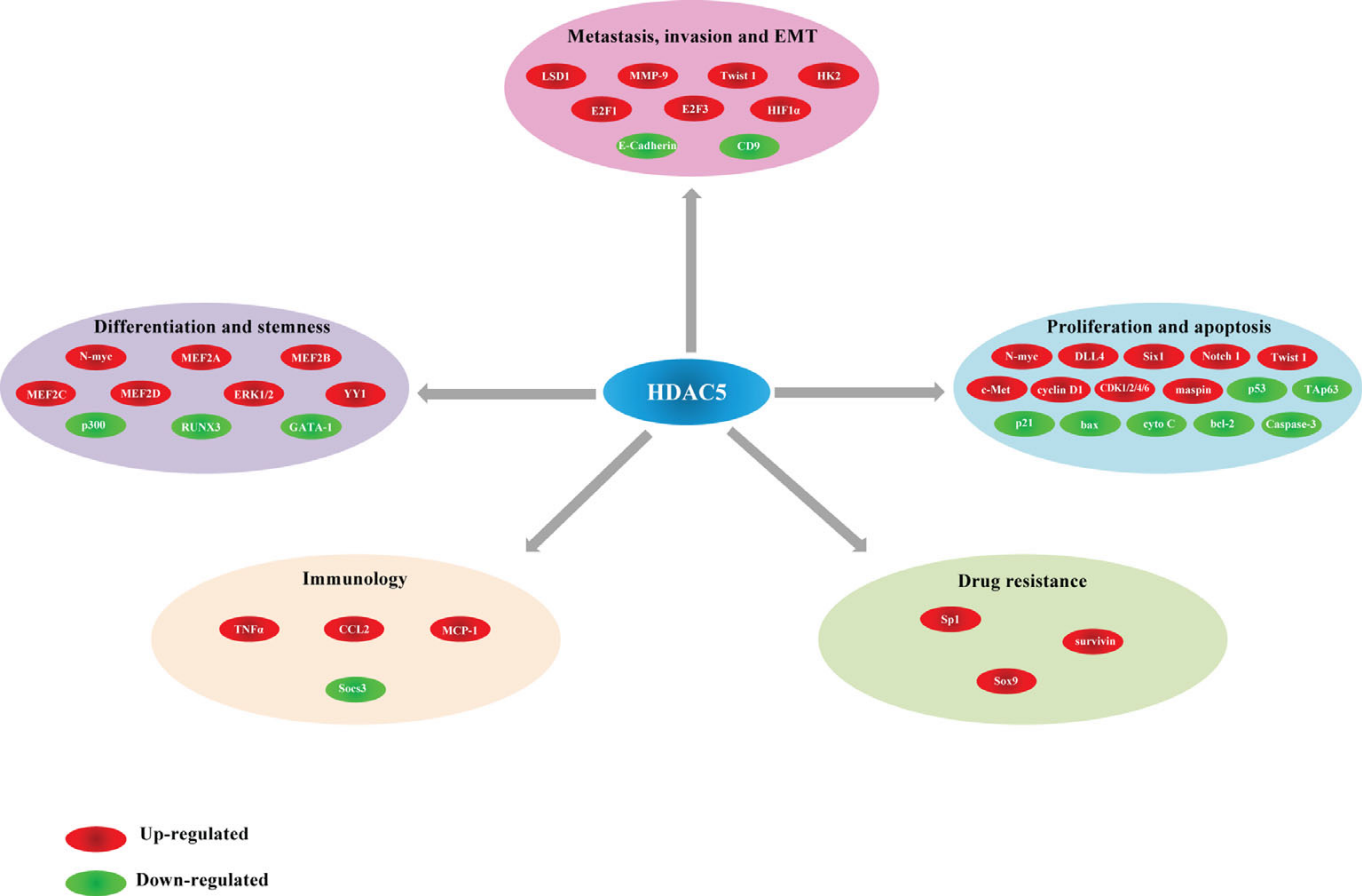
Different actions of HDAC5 and their corresponding target genes in cancer.

The functions of HDAC5 in tumorigenesis have been investigated in a variety of cancers. HDAC5 plays distinct roles in different cancer types. In this review, we summarize recent findings on the biological activities of HDAC5 in several common cancers and cancer-related processes (**Table 2**, **Figure 3**).

**Table 2:** Multiple cellular processes of HDAC5 in cancer management.

Tumor type	Expression status	Target genes	Effect upon cell lines	References
Breast Cancer	Up-regulation	LSD1	Increase cell metastasis and invasion	(50)
	Up-regulation	p53	inhibit cell proliferation	(51)
	Up-regulation	RUNX3	increase cell stemness	(46)
	Up-regulation	SOX9	increase chemoresistance	(52)
	Up-regulation	miR-125a-5p, Sp1, survivin	increase chemoresistance	(53)
Neuroblastoma	Up-regulation	CD9	Increase cell metastasis and invasion	(54)
	Up-regulation	N-myc	promote cell proliferation	(55)
	Up-regulation	N-myc	block cell differentiation	(55)
Medulloblastoma	Up-regulation	caspase-3	inhibit cell apoptosis	(56)
Lung Cancer	Up-regulation	DLL4, Six1, Notch1, Twist1	promote cell proliferation	(22)
Colorectal Cancer	Up-regulation	DLL4	promote cell proliferation	(57)
Osteosarcoma	Up-regulation	Twist	promote cell proliferation	(30)
	Up-regulation	N/A	maintain long telomeres’ length	(58)
Fibrosarcoma	Up-regulation	N/A	maintain long telomeres’ length	(58)
Wilms’ tumor	Up-regulation	c-Met	promote cell proliferation	(31)
Glioma	Up-regulation	Notch1	promote cell proliferation	(29)
Hepatocellular carcinoma	Up-regulation	Six1	promote cell proliferation	(59)
	Up-regulation	p21, cyclin D1, CDK2/4/6	promote cell cycle	(60)
	Up-regulation	p53, Bax, cyto C, caspase-3, Bcl-2	inhibit cell apoptosis	(60)
Urothelial Carcinoma	Down-regulation	TGF-β	hinder cell proliferation	(61)
Lymphoma	Up-regulation	TNF-α, MCP-1	induce pro-inflammatory function	(62)
Pancreatic Cancer	Up-regulation	Socs3, CCL2, TGF-β	promote macrophage recruitment	(63)
Ovarian Cancer	Up-regulation	YY1, miR-99a	increase cell stemness	(64)

### Role of HDAC5 in Cancer Metastasis and Invasion

PCR and immunohistochemical analyses have shown that HDAC5 was highly expressed in the cytoplasm of malignant epithelial cells, and HDAC5 expression was positively associated with distant metastasis and lymph node metastasis (65). HDAC5 expression was also found to be positively associated with intrahepatic metastasis and distant metastasis in HCC (21). In vitro and *in vivo* experiments have demonstrated that HDAC5 knockdown blocked metastasis of melanoma cells (28). Elevated HDAC5 expression is frequently observed in the luminal A and B subtypes of breast cancer, and HDAC5 silencing suppressed breast cancer cell motility and invasion (50). HDAC5 was also shown to promote cell invasion and metastasis in neuroblastoma (54), pancreatic cancer (66) and lung cancer (67).

In addition, HDAC5 enhanced the invasiveness of gastric cancer cell lines by stimulating protein kinase C (PKC)/matrix metalloproteinase 9 (MMP9) (25). Science MMP9 promotes tumor invasion and metastasis through extracellular matrix remodeling, regulation of cell adhesion, and degradation of vascular basement membrane and perivascular matrix during EMT (68, 69), the correlation between HDAC5 and EMT during tumorigenesis has drawn a great attention (**Table 3**).

**Table 3:** The involvement of HDAC5 in epithelial-mesenchymal transition (EMT) processes.

Tumor Type	Up/down regulated	Target Genes	References
Hepatocellular carcinoma	Up	HIF1α	(70)
	Up	Tbx3	(71)
Gastric cancer	Up	MMP9	(25)
Lung cancer	Up	E2F1, E2F3, Twist1	(27)
	Up	HK2	(72)
	Up	E2F1, E2F3, Twist1, MMP2, MMP9, Vimentin	(27)
Glioma	Down	E-cadherin, Vimentin	(73)
Urothelial Carcinoma	Down	Cytokeratin 5, E-Cadherin, Vimentin	(61)

In HCC, HDAC5 was shown to be involved in T-box 3 (Tbx3)-mediated EMT and metastasis, which was dependent on two HDAC5-interacting motifs (71). During HCC progression, application of the nonselective HDAC inhibitor (HDACi) panobinostat increased the expression of E-cadherin, an epithelial marker that is downregulated during EMT (74). HDAC5 was also found to promote doxorubicin-induced EMT in glioma cells, which could account for chemoresistance in glioma (73). On the other hand, it is worth noting that HDAC5 induced EMT but inhibited cell proliferation in urothelial carcinoma (UC) (61).

The basic helix-loop-helix transcription factor Twist1 can induce EMT by negatively regulating the transcription of E-cadherin (75). Twist1 is a downstream target of HDAC5 in osteosarcoma progression (30). In non-small cell lung cancer, HDAC5 activity was shown to induce the expression of EMT-related genes including the transcriptional regulators E2F1, E2F3, and Twist1 (27), providing evidence for its direct involvement in promoting EMT. As dysregulation of energy metabolism also contributes to EMT (76), it is possible that metabolic reprogramming is involved in HDAC5-mediated EMT. It was recently demonstrated that HDAC5 together with HDAC4 enhanced glycolysis by inducing the upregulation of hexokinase 2 (HK2), which was critical for EMT induced by hypoexpression of 5′ AMP-activated protein kinase (AMPK) in lung cancer (72).

### Role of HDAC5 in Cancer Proliferation and Apoptosis

The contribution of HDAC5 to cancer cell proliferation has been investigated in many studies. For example, HDAC5 overexpression was shown to promote cell growth, while small interfering RNA (siRNA)-mediated silencing of HDAC5 caused cell cycle arrest at the G0 phase in medulloblastoma (56). In neuroblastoma, HDAC5 promoted cell proliferation but had little effect on cell death (55). Overexpression of HDAC5 increased the proliferation of lung cancer cells, possibly *via* upregulation of downstream target genes (22). HDAC5 also increased DLL4 expression in colorectal cancer (CRC) cells, thereby enhancing their proliferation (57). HDAC5 induced osteosarcoma cell proliferation in a Twist1-dependent manner, highlighting the varied functions of the HDAC5/Twist1 axis in tumor progression (30). HDAC5 also increased c-Met expression to promote Wilms’ tumor cell proliferation (31). Notch1 signaling plays an important role in cancer cell proliferation (77, 78); HDAC5 was shown to promote Notch1 expression in glioma cells, leading to increased glioma cell proliferation (29). In HCC, HDAC5 activated cell proliferation by inducing Six1 expression, providing the first evidence for the oncogenic role of HDAC5 in HCC development and progression (59). However, HDAC5 also inhibits tumor cell proliferation. For example, elevated levels of HDAC5 in UC cells suppressed cell proliferation, an effect that may involve transforming growth factor β (TGF-β) (61).

Electron microscopy and immunohistochemical analyses of HDAC5 subcellular localization revealed that HDAC5 was associated with heterochromatin in S and G1 phases, suggesting a role in DNA replication and cell cycle progression (79). For example, HDAC5 silencing shielded cell-cycle of prostate cancer cells from RB-mediated repression (80). Knockdown of HDAC5 also induced G1 cell cycle arrest, which hindered breast cancer proliferation (50). Overexrpression of HDAC5 increased S phase of non-small cell lung cancer (NSCLC), which demonstrated that HDAC5 promote NSCLC proliferation through inducing DNA replication (27).

Cancer occurrence and progression are caused not only by abnormal cell proliferation and differentiation, but also by dysregulation of apoptosis. In fact, inducing cell apoptosis has become an important therapeutic strategy in cancer (81), and clarifying the role of cell apoptosis in cancer can provide insight into the molecular basis of tumorigenesis as well as a basis for the development of effective therapies. Recent studies have demonstrated the involvement of HDAC5 in cancer cell apoptosis. HDAC5 knockdown in medulloblastoma cell lines increased caspase-3 activity, which is important for apoptosis induction (56); and upregulation of apoptosis-related proteins and morphologic changes associated with apoptosis were observed in HeLa and MCF-7 cells transfected with a siRNA targeting HDAC5 (79). Overexpression of HDAC5 in HCC cells was correlated with reduced expression of p21 and hyperactivation of cyclin D1 and cyclin-dependent kinase 2/4/6 (CDK2/4/6), indicating that HDAC5 promotes cell cycle progression and blocks apoptosis in HCC tumorigenesis (60). However, in another study, overexpression of HDAC5 in MCF-7 breast cancer cells inhibited proliferation and promoted apoptosis in a p53-independent manner (51). Thus, HDAC5 may have opposite functions depending on the cellular context and interaction partner.

### HDAC5 and Immuno-Oncology

Malignant cells can escape immune surveillance and rapidly proliferate to form a tumor. HDACs have been implicated in the immune response (82–84), and the regulation of HDACs in cellular immunity plays an important role in tumorigenesis. HDAC5 interacts with the immune system—including immune cells and inflammatory cytokines—in cancer occurrence or progression. HDAC5 was shown to be involved in macrophage differentiation in lymphoma U937 cells (85), and HDAC5 depletion in U937 cells reduced the levels of tumor necrosis factor α (TNF-α) and monocyte chemoattractant protein 1 (MCP-1) *via* stimulation of NF-κB activity, suggesting a regulatory function for HDAC5 in the proinflammatory response of macrophages (62). In vitro and *in vivo* experiments have shown that the suppressor function of regulatory T cells (Tregs) was attenuated in HDAC5^−/−^ mice. Meanwhile, HDAC5 silencing suppressed the switch from CD4^+^ T cells to Tregs under polarizing conditions and inhibited interferon γ (IFN-γ) production by CD8^+^ T cells (86). Another study demonstrated that HDAC5 recruited macrophages to the tumor microenvironment through the Suppressor of cytokine signaling 3 (Socs3)/C-C motif chemokine ligand 2 (CCL2) axis and promoted pancreatic cancer *via* TGF-β-dependent paracrine signaling (63). These findings indicate that targeting HDAC5 is a promising cancer immunotherapy strategy.

### HDAC5 in Cancer Cell Differentiation and Stemness

Aberrant differentiation plays an important role in tumorigenesis. Epigenetic modifications including histone deacetylation are involved in cell differentiation (87). As such, clarifying the role and mechanisms of HDAC5 in cellular differentiation may provide a basis for new therapeutic strategies in cancer.

HDAC5 overexpression inhibited murine embryonic kidney(MEK) cell differentiation through transcriptional repression of GATA-1 and prevented the colocalization of the 2 proteins during erythroid differentiation (88). HDAC5 was also observed to block N-myc-mediated differentiation in neuroblastoma cells (55). MEF2 family proteins, which are recruited by HDAC5, include MEF2A, MEF2B, MEF2C, and MEF2D (89). Alternative splicing of MEF2 genes can yield protein isoforms with distinct functions (90). MEF2Cα2 engaged in a weaker interaction with HDAC5 than MEF2Cα1 in rhabdomyosarcoma (RMS) cell lines, which nonetheless enhanced the ability of the MEF2Cα2 isoform to promote RMS differentiation (91).

Cancer stem cells play a key role in tumorigenesis and can significantly influence the response to tumor therapy (92). HDAC5 increased the stemness of human breast cancer stem cells through inhibiting the binding of the Runt-related transcription factor 3(RUNX3)/p300 complex to the promoter of target genes (46). HDAC5 showed elevated expression in tumorspheres formed by H460 lung cancer cells. Application of the HDAC5 inhibitor LMK-235 reduced extracellular signal-regulated kinase 1/2 (ERK1/2) phosphorylation in a dose-dependent manner, demonstrating that the HDAC5-ERK1/2 axis plays an important role in maintaining the stemness of lung cancer stem cells (93). Additionally, during ovarian cancer (OC) progression, HDAC5 interacted with YY1 to promote OC cell stemness by deacetylating the promoter of the microRNA miR-99a (64). These results imply that HDAC5 regulates cancer cell differentiation and stemness through interaction with different cofactors.

### HDAC5 and Drug Resistance

Drug resistance is a major reason for cancer treatment failure. The main mechanisms of resistance are enhanced anti-apoptotic capacity, hyperactivation of cell proliferation, DNA replication, and cell cycle progression (94). Given its important role in cell proliferation, cell cycling, and apoptosis, HDAC5 may play a key role in the development of therapeutic resistance.

HDAC5 knockdown was shown to increase the sensitivity of MCF-7 and HeLa cells to doxorubicin and cisplatin by inducing heterochromatin decondensation (79). HDAC5 was illustrated to be involved in sorafenib resistance of HCC as well (95). Tamoxifen is a first-line treatment for breast cancer, HDAC5 deacethylated SOX9 and made it localized in the nucleus, which was responsible for tamoxifen resistant in breast cancer (52). HDAC5 expression was also elevated in estrogen-independent breast cancer cells and induced tamoxifen resistance *via* the miR-125a-5p/specificity protein 1 (Sp1)/survivin axis (53). Additionally, formononetin inhibited HDAC5 expression to attenuate doxorubicin-induced EMT, thereby promoting doxorubicin sensitivity in glioma cells (73).

Telomeres are small DNA-protein complexes located at the end of linear chromosomes in eukaryotic cells that maintain chromosome integrity and are involved in cell cycle regulation (96). During tumorigenesis, telomerase stimulates the re-synthesis of telomeric DNA, allowing cancer cells to proliferate continuously, which reduces the efficacy of chemotherapy (97). Telomeres are subject to various epigenetic modifications (98–100). HDAC5 preferentially localized at long telomeres and maintains their length, and HDAC5 silencing was found to increase the sensitivity of osteosarcoma and fibrosarcoma cells with long telomeres to chemotherapy (58).

## HDAC5 and Oncogenetic Signaling Pathways

**Figure 4:**
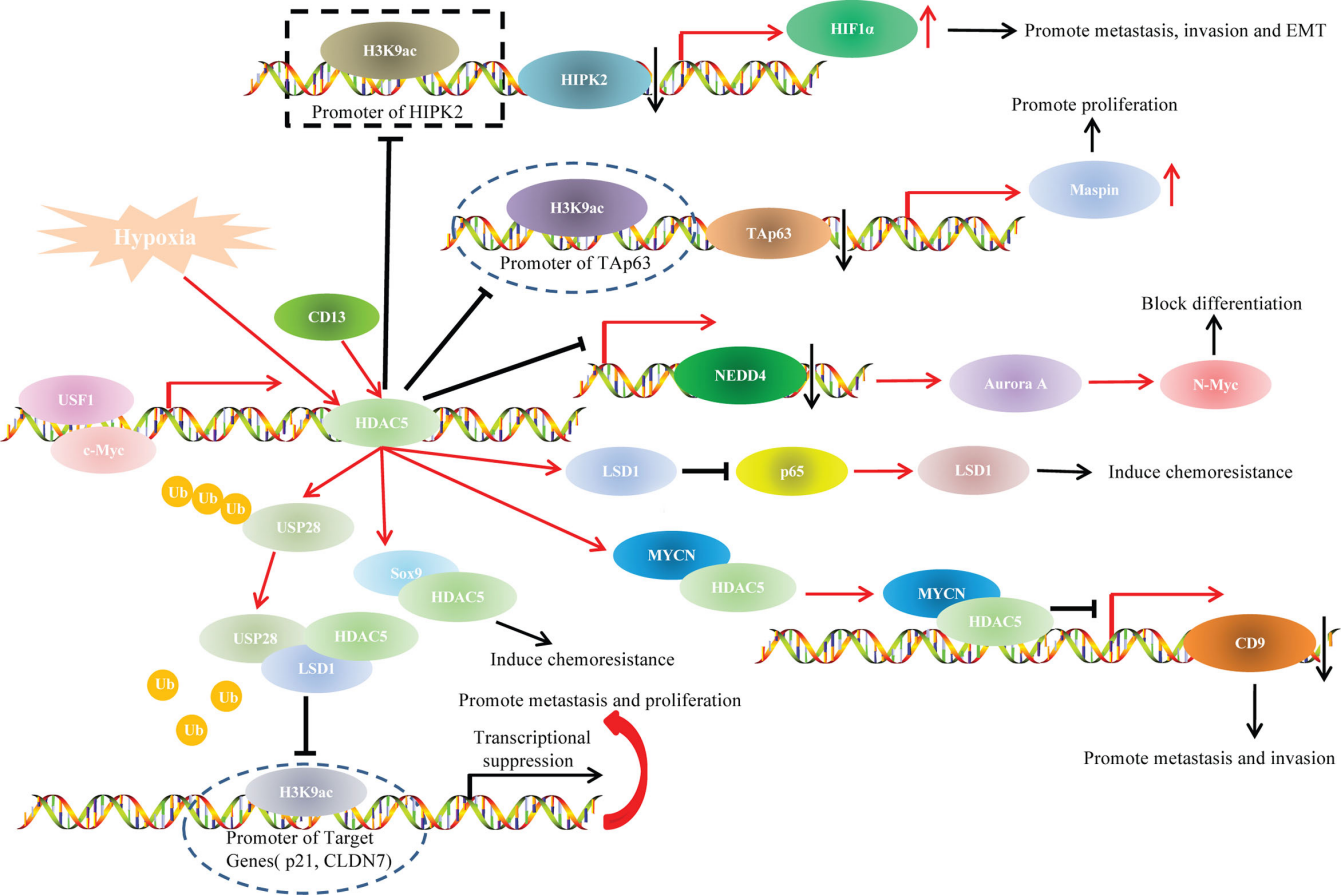
DAC5 and oncogenic signaling pathways.

Studies have indicated that HDAC5 participated in cancer progression through various oncogenetic signaling pathways, such as TAp63/maspin (21), HIPK2/HIF1α (70), and p65/NF-κB pathways (95)(**Figure 4**).

### HDAC5 and TAp63/maspin Signaling

P63 is a new member of the p53 gene family. Although its structure is highly homologous to p53, p63 encodes a special C-terminal sequence, which contains sterile α group and a transcriptional inhibition domain. The C-terminus can bind to lipid membranes and inhibit transcription (101). Up to 14 p63 homologs have been identified, which are produced by transcription from different promoters and selective cleavage. The three p63 homologs that are produced through alternative splicing of mRNA transcribed from the promoter upstream of intron 1 are named TAp63α, TAp63β and TAp63γ because they contain a transcription domain (TA) (102). Many studies have demonstrated that these homologs are important transcriptional activators of mammary serine protease inhibitor (Maspin) (103, 104). Maspin (SERPINB5), a member of the serine protease inhibitor superfamily, is not only a serine protease inhibitor gene, but also a new soluble protein that is found in the cytoplasm. It can inhibit angiogenesis, increase cell adhesion, induce apoptosis and inhibit growth and metastasis of cancer cells (105, 106).

In our previous study, we explored the role of HDAC5 in HCC and found that HDAC5 overexpression induced HCC proliferation and tumorigenesis *in vitro*. We also knocked down HDAC5 in Hep3B (p53-null) and HepG2 (p53-WT) cells and investigated the expression levels of p53 family members. The results indicated that HDAC5 downregulated p53, TAp63, and p73 at both the mRNA and protein levels. In addition, TAp63 gradually decreased with HDAC5 overexpression in immortalized liver cell line (THLE-3 cells). Chromatin immunoprecipitation (ChIP) showed that HDAC5 knockdown increased the acetylation at the TAp63 promoter (i.e., at Lys9 of histone H3 [H3K9ac]), which indicated the suppressive effect of HDAC5 on TAp63 transcription. Rescue experiments showed that HDAC5 promoted proliferation and tumorigenesis *via* the Tap63/Maspin axis. In summary, our findings demonstrated that HDAC5 knockdown increased acetylation at the TAp63 promoter, resulting in TAp63 hyperactivation and Maspin overexpression, which blocked HCC cell proliferation and tumorigenesis (21).

### HDAC5 and HIPK2/HIF1α Signaling

Hypoxia plays an important role in tumorigenesis and metastasis. Hypoxia inducible factor 1 (HIF1) is a key transcription factor that can transmit the hypoxia signal and mediate the effect of hypoxia. HIF1α is a functional subunit of HIF-1. Under hypoxia, HIF1α activates downstream target genes through interacting with hypoxia-response elements, which thereby regulate proliferation, apoptosis, invasion, metastasis, angiogenesis, energy metabolism, and chemoradiotherapy resistance of cancer cells (107).

Epithelial-to-mesenchymal transition (EMT) is a process that occurs under normal physiologic conditions such as embryonic development and wound healing. During EMT, epithelial cells lose their polarity and acquire mesenchymal-like features along with the capacity to migrate and invade other tissues (108). An increasing number of studies have reported that the change in the oxygen level in cancer microenvironments and the HIF1α-induced hypoxia signal transduction pathway acted as a vital regulator of EMT, which played a key role in hypoxia-induced cancer invasion and metastasis (109, 110).

In our previous study, we illustrated that HDAC5 induced HCC migration, invasion and EMT only under hypoxia but not under normoxia. Although a dual luciferase reporter assay indicated increased transcription of HIF1α after ectopic HDAC5 expression under hypoxia, HDAC5 did not transactivate HIF1α directly even if under hypoxia. Further mechanistic analysis using ChIP assays unveiled that, under hypoxia, HDAC5 was recruited to the HIPK2 promoter; HDAC5 knockdown increased H3K9ac modification at this promoter, and the resultant upregulated HIPK2 bound to the HIF1α promoter to inhibit HIF1α expression. Our findings revealed that the HDAC5/HIPK2/HIF1α axis contributed to hypoxia-induced aggressiveness of HCC (70).

### HDAC5 and LSD1 Signaling Pathways

Lysine-specific histone demethylase 1 (LSD1), also known as KDM1A or AOF2, is the first identified histone specific demethylase (111). LSD1 exhibits varying functions related to cancer progression in different cancer types, as the LSD1 protein is methylated in some cancer type and demethylated in others (112). HDAC5 was positively associated with LSD1 levels in breast cancer cells and tissue specimens. In vitro experiments unveiled that HDAC5 promoted invasion, metastasis and tumorigenic development *via* LSD1. Co-immunoprecipitation (Co-IP) and deletion mapping studies demonstrated that the NLS element was responsible for the HDAC5-LSD1 interaction. Protein ubiquitination assays indicated that HDAC5 stabilized LSD1 through blocking USP28 polyubiquitination. These results suggest an important role for the HDAC5/LSD1 axis in tumorigenesis (50).

In addition, HDAC5 also functioned as a downstream target gene of various upstream factors and participated in the regulatory pathways during cancer progression. For example, USF1 transactivated HDAC5 by physically binding to the HDAC5 promoter at −356 to −100 bp, thereby increasing the stability of LSD1 to induce chemoresistance of breast cancer (113).

### HDAC5 and p65/NF-κB Signaling

NF-κB is a transcription factor involved in cell proliferation and transformation, apoptosis, immune responses and other important biological activities (114). NF-κB protein usually forms homodimer/heterodimer from p65 and p50, and is inactivated in cytoplasm due to the formation of trimer complex with inhibitor IKB (115). Therefore, the correlation between HDAC5 and p65/NF-κBsignaling during tumor progression has drawn researchers’ attention. Hu et al. illustrated that CD13 promoted sorafenib resistance in HCC *via* indirectly increasing p65 protein stability. Co-IP indicated a strong interaction between CD13 and HDAC5. Truncation assays demonstrated that the N-terminal region of HDAC5 protein (amino acids 1-684), and the N-terminal cytoplasmic domain of CD13 (amino acids 2-8) accounted for the HDAC5-CD13 interaction. Hence, CD13 interacted with HDAC5 to increase the stabilization of LSD1, which demethylated p65 (50, 116) and thereby stabilized it and increased p65/NF-κB signaling; this resulted in sorafenib resistance and HCC progression (95).

### HDAC5 and Myc Family

The Myc gene family is one of the most widely studied family of nuclear oncogenes. This family includes three main members: c-myc, N-myc and L-myc. N-myc protein can be stabilized with phosphorylated at serine 62 (Ser62) or be degraded with phosphorylated at threonine 58 (Thr58) (117). HDAC5 knockdown reduced N-myc at protein levels but had little effect on N-myc mRNA levels, so HDAC5 upregulated N-myc at the posttranscriptional level. HDAC5 overexpression significantly upregulated total N-myc protein in cells with Ser62 mutant N-myc but not in cells with Thr58-mutant N-myc. This indicated that HDAC5 upregulates N-myc protein by increasing N-Myc protein stability. Microarray and ChIP assays revealed that HDAC5 knockdown led to the transactivation of NEDD4 (an E3 ubiquitin–protein ligase). This illustrated that HDAC5 stabilized N-myc protein through suppressing the transcription of NEDD4 (55). Interestingly, HDAC5 was also shown to interact with N-myc protooncogene protein (MYCN), and the complex colocalized to the CD9 promoter and attenuated CD9 expression in neuroblastoma cells, leading to tumor cell invasion and metastasis (54).

In addition, C-myc transactivited HDAC5 though directly binding to the −200/+20 bp region upstream of the transcription start site of HDAC5, which contained C-myc-specific binding sequence (CACGTG). In turn, HDAC5 promoted SOX9 nuclear localization *via* interaction with its HMGB domain (amino acids 1–181) in estrogen receptor-positive breast cancer cells. These results indicated that the C-myc/HDAC5/SOX9 axis is a potential target for estrogen receptor-positive breast cancer therapy (52).

## Clinical Significance of HDAC5 in Cancer

### HDAC5 and Cancer Diagnosis

Cancer cells release proteins into the peripheral blood during tumorigenesis. Thus, circulating proteins can serve as prognostic biomarkers for predicting disease recurrence and informing treatment decisions (118). Circulating HDAC5 is a potential marker for cancer diagnosis, given that it is detected at different levels in the peripheral blood of cancer patients as compared to healthy subjects or patients with nonrecurrent disease. One study found that the specificity of HDAC5 in distinguishing CRC patients from healthy subjects was 96.3%, indicating that it can be a diagnostic biomarker for CRC (32). Proteomic analysis with an antibody array identified proteins that were differentially expressed between breast cancer patients with and those without recurrence (34); moreover, HDAC5 was detected at a significantly higher level in the blood in patients with recurrent breast cancer than in those with recurrent triple-negative breast cancer.

Tumor antigens can provide insight into antitumor immune responses and the mechanisms of immune escape used by cancer cells, which can aid cancer diagnosis and the establishment of immunotherapy strategies. An antibody reactivity screen revealed HDAC5 as the only serum antigen that differed between CRC patients and healthy subjects (33). Thus, serum HDAC5 has clinical utility as a diagnostic biomarker for cancer.

### HDAC5 and Cancer Therapy

#### HDAC5 and HDAC Inhibitors

Molecular targeted therapies have specific antitumor effects, which limits their toxicity. Some studies demonstrated that HDAC5 is a potential therapeutic target for HDAC inhibitors (HDACis) as anticancer drugs (17, 25, 46, 73, 74, 119–123)(**Table 4**). HDACis can be divided into 4 groups according to their chemical structure—namely, hydroxamic acids, benzamides, cyclic peptides, and carboxylic acids. The basic structural features of HDACis are a hydroxamic acid complexed with Zn^2+^, intermediate fatty chain linker, and lipophilic cap that engages in hydrophobic interaction with the binding pocket (127). HDACis could be divided into pan-HDACis and selective class IIA HDACis.

**Table 4:** Summary of HDAC5-targeted drugs in cancer therapy.

Agents	Anti-tumor properties	Reference
Trichostatin A (TSA)	Inhibit proliferation of gastric cancer	(25)
	Reduce chemoresistance of cancer	(120)
	Inhibit proliferation of breast cancer	(124)
	Induce apoptosis in breast cancer	(46)
Belinostat (PXD101)	Block chemoresistance in estrogen receptor positive breast cancer	(119)
Sodium butyrate	Inhibit migration and EMT in HCC	(71)
Panobinostat (LBH589)	Suppress invasion and metastasis in neuroblastoma	(54)
	Decrease cell viability and proliferation declined,and increase apoptosis and autophagy in HCC	(74)
Vorinostat	Reduce chemoresistance of cancer	(120)
Luotonin-A	Cause apoptosis and senescence in Hela cells	(17)
Formononetin	Prevent EMT in glioma	(73)
LMK-235	Reduce chemoresistance of cancer	(120)
	Induce apoptosis of pNET	(125)
	Induce apoptosis and reduce proliferation and migration of breast cancer	(20)
	Inhibit proliferation, metastasis and invasion of breast cancer	(65)
	Inhibit stemness of lung cancer	(93)
AR-42	Induce apoptosis in HCC	(121)
Vorinostat (SAHA)	Induce apoptosis in breast cancer	(122)
Ebselen	Decrease cell viability of cancer cells	(123)
Sulforaphane	Inhibit proferation of breast cancer	(113)
Simvastatin	Suppress proliferation of pancreatic cancer	(66)
	Inhibit proliferation of CRC	(126)

Trichostatin A (TSA)is the first identified pan-HDACis with an inhibitory activity depends on Zn^2+^-dependent HDAC enzymes. The crystal structure of TSA and HDAC analogues showed that the conserved deacetylase active center were made up by a tubular bag, a zinc binding region and two ASP his (aspartate histidine) charge relay networks. The long fatty chain of TSA bound to the inner part of the tubular bag, and the hydroxime group at the end of the fatty chain showed connection with the zinc binding region through carbonyl and hydroxyl groups, which thereby inhibiting the ability of HDACs including recognizing substrate, regulating enzymatic activity and interacting with other proteins (128). TSA was illustrated to inhibit HDAC5 to block malignant behavior of cancer cells, including chemoresistance (120), proliferation (124) and invasion (25). Other HDACis such as belinostat, givenostat, panobinostat and dacinostat have the same mechanism with TSA as well. Among them, belinostat(PXD101) and panobinostat(LBH589) were US FDA-approved for cancer therapy (129, 130). Belinostat has been revealed to attenuate chemoresistance in estrogen receptor positive breast cancer *via* targeting class IIA HDACs including HDAC5 (119). Besides, HDAC3 and HDAC5 had copy number gain during HCC progression, panobinostat might inhibit HDAC3 and HDAC5 to decrease proliferation and induce apoptosis and autophagy of HCC, and this anticancer effect could be augmented by combination therapy with panobinostat and sorafenib (74).

Vorinostat(SAHA) is one of HDACis approved by FDA for cancer therapy. The hydroxime group of SAHA, also bound to Zn^2+^ in the way similar with TSA, but the fatty chain of SAHA showed less connection to the tubular bag than that of TSA, which indicated that the inhibitory activity of SAHA is lower than TSA (131). Hence, combination therapy with SAHA and other therapeutic drugs has drawn researchers’ attention. Vasilatos et al. (122) explored the role of pargyline (an LSD1 inhibitor) and SAHA in breast cancer, the results demonstrated that both pargyline and SAHA suppressed cell proliferation and induced apoptosis through inhibiting HDAC5. Meanwhile, combination therapy with pargyline and SAHA led to superior anticancer effect. Interestingly, this anticancer effect was not found in non-TNBC counterparts or non-tumorigenic breast cells, which illustrated therapeutic efficacy of SAHA on breast cancer was subtype-dependent.

LMK-235, a selective HDACi showed preferable interactions with the catalytic zinc ion of class IIA HDACs, was illustrated to decrease phosphohistone H3 and Ki-67 levels in pancreatic neuroendocrine tumors (pNETs) while inducing pNET cell apoptosis and histone H3 acetylation, making it an effective targeted therapy for pNET that indirectly antagonizes the activity of HDAC4/5 (125). Similar findings were reported in breast (20, 65) and lung (93) cancers. However, LMK-235 induced HDAC5 expression in UC cells, indicating that HDAC5 is not a suitable therapeutic target in UC (132). Cao et al. (113) selected a series of HDACis including SAHA, TSA, LBH-589, PXD-101, MS-275, MC-1568, Romidepsin and Sulforaphane (SFN) to test their inhibitory effect towards HDAC5 expression on breast cancer cells. The results illustrated that SFN showed most significant inhibitory effect on HDAC5 expression at both mRNA level and protein level. Dual luciferase reporter assay indicated that SFN hindered the transcriptional activity of HDAC5 *via* affecting the region from −356 to −100 bp at HDAC5 promoter. In addition, USF1 could block the downregulation of HDAC5 mediated by SFN. These finding suggested SFN as a potential drug for breast cancer therapy.

#### HDAC5 and Metabolism Inhibitors

Hendrick et al. (133) demonstrated that knock down of HDAC5 induced cancer apoptosis through an iron-dependent reactive oxygen species (ROS) production. In addition, HDAC5-knock down cells adapted oxidative stress through glucose and glutamine metabolic reprogramming. Therefore, blocking glucose and glutamine metabolism in HDAC5-knock down cancer cells significantly induced cell death, which provided insight into a combination therapy with HDAC5 inhibitors and various inhibitors of metabolism as a new strategy for cancer treatment.

During tumorigenesis, cancer cells often undergone deregulated lipid metabolism (134). Statins, a class of 3-hydroxy-3-methylglutaryl coenzyme A (HMG-CoA) reductase inhibitors, can modulate the cell cycle, signal transduction pathways, and angiogenesis, and thus have therapeutic potential in cancer treatment (135–139). Simvastatin was shown to attenuate pancreatic cancer growth by inhibiting the oxysterol binding-related protein 5 (ORP5)/HDAC5 axis (66). Additionally, the combination of statins and other therapeutics was shown to prolong survival in cancer patients (140). For example, statins inhibited CRC cell proliferation by modulating the expression of enhancer of zeste homolog 2 (EZH2) and HDAC5, an effect that was enhanced in the presence of MC-1568, a class II HDACi. The underlying mechanism involved the upregulation of p27^(KIP1)^ by statin *via* inhibition of EZH2; MC-1568 further increased p27^(KIP1)^ expression by suppressing HDAC5, resulting in an antiproliferative effect in CRC (126).

Collectively, these findings highlight the clinical significance and utility of HDAC5 in targeted cancer therapy.

## Conclusion

Accumulating evidence indicates that HDAC5 can serve as a biomarker for tumorigenesis in a variety of cancer types. HDAC5 expression is correlated with clinicopathologic features of cancer patients, highlighting its clinical value. Elucidating the targets and mechanisms of action of HDAC5 will enhance our understanding of the molecular basis of tumorigenesis and provide novel markers for early diagnosis, treatment response monitoring, and predicting disease prognosis, as well as an empirical basis for the development of effective cancer treatments.

## Author Contributions

XX and MY contributed to the study conception and design. XX edited the manuscript language. JY and CG wrote the main manuscript text and prepared the figures and tables. QK, ZF, and XC provided advice regarding the manuscript. All authors contributed to the article and approved the submitted version.

## Conflict of Interest

The authors declare that the research was conducted in the absence of any commercial or financial relationships that could be construed as a potential conflict of interest.

## Supplementary Material

Supplementary Table 1 microRNAs predicted to regulate HDAC5.
